# Assessment of Aerobic Capacity and Other Cardiopulmonary Parameters in Children with Juvenile Idiopathic Arthritis

**DOI:** 10.3390/biomedicines13112672

**Published:** 2025-10-30

**Authors:** Aleksandra Stasiak, Piotr Kędziora, Aleksandra Ryk, Jerzy Stańczyk, Elżbieta Smolewska

**Affiliations:** 1 Department of Pediatric Cardiology and Rheumatology, Medical University of Lodz, 91-738 Lodz, Poland; 2 Department of Biostatistics and Translational Medicine, Medical University of Lodz, Mazowiecka 15 Street, 92-215 Lodz, Poland

**Keywords:** JIA, cardiopulmonary exercise test, child

## Abstract

**Introduction:** Juvenile idiopathic arthritis (JIA) is the most common chronic rheumatic disease in children. It is believed that children with JIA have lower cardiopulmonary capacity and worse exercise tolerance. The gold standard for assessing physical fitness is aerobic fitness, commonly referred to as the maximum or peak oxygen uptake volume (peakVO_2_) measured during a maximum load exercise test. Reduced aerobic fitness may play a key role in predicting the health of JIA patients as it has been associated with cardiovascular diseases and increased adult mortality. **Methods:** The aim of this study was to assess the oxygen capacity of adolescents with JIA along with other cardiopulmonary parameters in order to determine a group of patients with increased risk of developing cardiovascular diseases in comparison with healthy individuals. Patients were assessed based on parameters such as age, sex, type of JIA, laboratory parameters, physical activity, and treatment. **Results:** Patients with JIA had lower median values of peakVO_2_ (29.05 vs. 38.02 mL/min/kg, *p* < 0.001), as well as other crucial cardiopulmonary parameters, such as O_2_ pulse, minute ventilation, oxygen uptake efficiency slope, and cardiac output than in the healthy control group. The ventilatory anaerobic threshold was achieved earlier and at lower VO_2_ values in children with JIA (*p* = 0.0001). Children with JIA also had lowered respiratory parameters such as maximal voluntary ventilation (*p* = 0.0031) and tidal volume (*p* = 0.0002). Patients who were physically active (moderate-intensity physical activity lasting at least 60 min per day) had significantly higher peakVO_2_ (*p* = 0.0099) and ΔVO_2_/ΔWR relationship (*p* = 0.0041) values than JIA patients who were not physically active. **Conclusions:** Children with JIA show moderate to severe physical impairment. Reduced physical fitness and a low level of activity might be associated with further deterioration of patient’s condition, which might contribute to increased risk of cardiovascular disease, social exclusion and deterioration of quality of life in this group of patients. Exercise programs that improve aerobic fitness and increase muscle strength should be individualized and modified based on the individual needs and capabilities of the patient.

## 1. Introduction

JIA is the most common chronic rheumatic disease in children [1]. JIA is a disease with a multifactorial and complex pathogenesis that includes environmental factors and genetic predisposition, among others. Development of the disease is associated with inflammatory and autoimmune processes, and its course is characterized by periods of exacerbation and remission [1,2]. The severity and duration of symptoms depend on the number and type of affected joints, pain, functional limitations and possible deformities that may occur in the subsequent stages of the disease. Children and adolescents with JIA may experience significant short- and long-term disability due to muscle weakness, joint pain, contractures and limited mobility. This disability can affect school performance, physical activity, family life and interactions with peers [3]. Patients with JIA are at risk of significant deterioration in their health-related quality of life compared to their healthy peers [4]. Treatment involves nonsteroidal anti-inflammatory drugs (NSAIDs), systemic and topical glucocorticosteroids, disease-modifying antirheumatic drugs (DMARDs), of which methotrexate is most commonly applied in children and adolescents, while in recent years the use of biological therapy has also become common [1,5]. It is also worth mentioning the development of nanomedicine in the treatment of rheumatoid arthritis (RA) and, in the future, probably JIA. Nanomedicine focuses on molecular mechanisms and provides targeted treatment focused on sites of inflammation with minimal side effects, thereby also supporting the cardiovascular system of RA patients by reducing disease activity and complications of the treatment itself [6]. Some of the targeting agents applied in the treatment of JIA include biologic drugs targeting specific pro-inflammatory cytokines, immune cells or osteoclasts. Particles applied as a targeting nanomedicine in the treatment of RA include coated ligand nanoparticles targeting macrophages and monocytes, gold nanoparticles attracting pro-inflammatory molecules, coated nanoparticles with ligand for synoviocytes target and coated nanoparticles for targeting endothelial cells. The limitations of this treatment method mainly concern the clinical translation of this therapy for the patient, the costs of producing these molecules, and their complexity [7,8].

In recent years, much has been said about aerobic fitness as a marker of cardiovascular morbidity and mortality [1]. Despite advanced therapeutic options, children with JIA have lower muscle strength, aerobic and anaerobic capacity, bone mineral density and decreased mobility. Due to pain, physical limitations and anxiety they less frequently participate in recreational activities and exhibit lower functional capacity. Disability and physical impairment resulting from low physical activity in the JIA patients may increase cardiovascular mortality in this group in adulthood [2]. It is believed that children with JIA have lower cardiopulmonary capacity and worse exercise tolerance. The gold standard for assessing physical fitness is aerobic fitness, commonly referred to as the maximum or peak oxygen uptake volume measured during a maximum load exercise test. Aerobic capacity is defined as the ability to perform dynamic exercise of moderate to high intensity involving large muscle groups for extended periods of time. It is an important determinant of overall health, with higher aerobic capacity being associated with lower morbidity and mortality. For children and adolescents with a chronic disease, such as JIA, maintaining or increasing aerobic capacity is important as it is inversely related to cardiovascular risk factors [9]. Peak oxygen uptake (peakVO_2_) achieved during a graded maximal-load exercise test is considered by the World Health Organization (WHO) to be the best indicator of aerobic capacity and a measure of cardiorespiratory fitness. It includes an assessment of the body’s oxygen transportation system during exercise and the integrated function of the pulmonary, cardiovascular and muscular systems [10,11,12]. Children with JIA show moderate to severe impairment of peakVO_2_ compared to healthy children, indicating a clinically significant reduction in physical capacity [13]. Aerobic capacity can be accurately assessed during a cardiopulmonary exercise test (CPET) [9].

## 2. Materials and Methods

The main aim of the study was to assess aerobic capacity and its relationship with disease activity parameters and their impact on the cardiopulmonary capacity of children and adolescents with JIA compared to healthy children. Specific objectives included assessment of: the correlation between aerobic capacity and inflammatory markers (C reactive protein—CRP; erythrocyte sedimentation rate—ESR); the correlation between aerobic capacity in JIA patients and the subtype of the disease; the correlation between aerobic capacity in JIA patients and the duration of the disease; the impact of the applied treatment (including biological treatment) on aerobic capacity; comparison of ventilatory parameters and gas exchange parameters between JIA patients and the control group; comparison of ventilatory and gas exchange parameters between physically active JIA patients and sedentary JIA patients.

The study consisted of 50 patients with JIA and 50 healthy individuals who served as a control group. During routine follow-up hospital visits, children with JIA underwent laboratory tests to assess the inflammatory activity of the disease. All laboratory tests were performed using standard methods. Immunological and genetic test results (presence and titer of ANA, RF, anti-CCP and HLA-B27 antibodies) as well as data on the subtype of the disease were obtained from the patients’ medical records. In order to assess children’s health and the severity of the symptoms of the disease, publicly available questionnaires (Child Health Assessment Questionnaire (CHAQ), Visual Analogue Scale (VAS)), containing questions about the child’s general health, daily physical activity, and a scale assessing joint pain were prepared for patients. The CHAQ questionnaire concerns the patient’s activity over the last 14 days and covers eight different categories (dressing, eating, walking, getting up, reaching, grasping, hygiene, and activity). Each question is graded on a scale of 0 to 3 points (0 = no difficulty, 1 = some difficulty, 2 = considerable difficulty, 3 = inability to perform the task). The total score ranges from 0 (no limitations) to 3 (significant limitations). The data supplemented by the physician included the number of joints affected by active inflammation and an assessment of the severity of the inflammatory process in the joints affected by the disease. Disease activity was assessed based on the CHAQ questionnaire completed by the patient, the Juvenile Arthritis Disease Activity Score-27 (JADAS-27), as well as laboratory tests (markers of inflammation—CRP, ESR) and joint ultrasound with assessment of joint involvement—Power Doppler Ultrasonographic Signal (PDUS scale. Patients with JIA underwent an ultrasound examination of the joints affected by the disease. The examination was performed prior to the exercise test, along with an assessment of inflammation and synovial vascularization using Power Doppler Ultrasound (PDUS). Patients were evaluated by an experienced pediatric rheumatologist specializing in ultrasound joint assessment (blinding was not applied, however the examinations were performed by the same person, who assessed these patients previously during the course of the disease, which guaranteed repeatability of the ultrasounds and the qualifications of the person performing the ultrasound were assessed beforehand). The affected joints were assessed on a 4-point scale, where 0 = no vascularization, 1 = mild (presence of single/vessel dots), 2 = moderate (presence of confluent vessel dots in less than half of the synovial area), and 3 = marked (presence of confluent vessel dots in more than half of the synovial area. Clinical parameters, such as patient’s age, sex, body mass index (BMI), number of affected joints, JIA subtype, applied treatment and its duration, and physical activity of the child were also taken under consideration. Regarding physical activity, patients completed a questionnaire consisting of four questions: 1. Do they perform ≥ 60 mins of physical activity every day during the last three months? 2. Is this intense or moderate activity (cycling, swimming, running, dancing, brisk walking—please specify the activity if it is other than those listed)? 3. Do they attend physical education classes? 4. If the patient answered “no” to the above questions, do they associate their limited physical activity with their illness? If the answer to the first three questions was positive, the patient was classified as physically active; if not, they were classified as physically inactive.

Subsequently, patients underwent a cardiopulmonary exercise test on a treadmill with appropriate load. During the test, both gas exchange and ventilation parameters were assessed. Heart rhythm was continuously monitored throughout the test using a 12-lead electrocardiogram (ECG). ECG recording was performed at rest in a horizontal position, then in an upright position, during every minute of exercise, and during a six-minute period of recovery. Blood pressure was measured using the oscillometric method at rest and every 3 min during exercise, as well as in the recovery phase. The patient was considered to have reached maximum effort if they met one of the following criteria:

1. RER was ≥1.00,
2. Heart rate during exercise was above 85% of the patient’s maximum heart rate calculated using a formula adjusted for the patient’s body weight,
3. Patient reached the predicted peak oxygen uptake and/or a VO_2_ plateau was observed,
4. Patient reported maximum fatigue, i.e., scored 8 or 9 on the 10-point Borg scale.

According to Bongers et al., additional “objective” physiological criteria for maximum effort during pediatric CPET are achieving adequate HR and RER values at VO_2_max [9]. More specifically, it is recommended to use HR of at least ≥95% of 195/min and RER of at least ≥1.00 at VO_2_max as additional criteria for maximum exertion during CPET in pediatric populations. The American Thoracic Society also states that although no RER value defines maximum effort, values greater than 1.15 are associated with near-maximum or maximum effort [14]. Amedro et al. in their study on reference values for cardiopulmonary exercise test parameters in the contemporary pediatric population also report maximal RER values of 1.15  ±  0.11 [15].

Peak VO_2_ was determined based on exhaled gas measurements during the exercise test. Exhaled gases (oxygen and carbon dioxide) were analyzed breath by breath using a continuous measurement method with each breath calculated using an Ergostik probe (Reynolds Medical, Warsaw, Poland) and Blue Cherry software (version 1.3.4.4, Geratherm Respiratory, Bad Kissingen, Germany)). Oxygen concentration was analyzed by an electrochemical method, while carbon dioxide was analyzed by infrared absorption. Before each test, the device was calibrated (gas and volume calibration) using standard gases with known oxygen and carbon dioxide concentrations. The highest VO_2_ achieved during the test, measured over a period of 10 s, was considered peakVO_2_. Other cardiopulmonary exercise test parameters assessed during the test were: maximum speed (km/h), maximum treadmill elevation (%), load (W), metabolic equivalent (METS), predicted peakVO_2_ (%), oxygen uptake (VO_2_; L/min), carbon dioxide excretion (VCO_2_; L/min), respiratory exchange ratio (RER), oxygen pulse (O_2_ pulse; mL/heartbeat), minute ventilation (VE; L/min), minute ventilation in relation to carbon dioxide emission and its slope (VE/VCO_2_; (VE/VCO_2_; VE/VCO_2_ slope), anaerobic ventilation threshold (VAT), respiratory compensation point (RCP), relationship between VO_2_ and work performed per unit of time (VO_2_/WR; mL/min/W), oxygen uptake efficiency index (OUES), cardiac output (CO; L/min), resting and maximum heart rate (HR), and heart rate reserve (HRR). Regarding respiratory parameters assessed during cardiopulmonary exercise testing, additional parameters were taken into account, including: breathing reserve (BR; L/min and %), respiratory rate (Bf), maximal voluntary ventilation (MVV; L/min), respiratory power (VP; mmHg), tidal volume (Vt; L), end-tidal partial pressure of oxygen (PETO_2_), and end-tidal partial pressure of carbon dioxide (PETCO_2_). Exercise test was performed on a medical diagnostic treadmill (GE Healthcare T2100, GE MEDICAL SYSTEMS POLSKA SP. Z O.O, Warsaw, Poland). Test was conducted according to Bruce protocol, which consists of several 3-min stages of exercise. At each stage, the incline and speed of the treadmill are increased. Stage 1 of the Bruce protocol is performed at a speed of 2.7 km per hour and an incline of 10%. In stage 2, the speed is 4.0 km per hour and the incline is 12%, and in stage 3, the speed is 5.5 km per hour with an incline of 14%. Before and after the test, the patient was asked about their pain in the joints affected by the disease. The data obtained during the study was recorded in databases, upon which, after the completion of the project, statistical analysis including correlations between the parameters obtained in the cardiopulmonary exercise test and the results of laboratory tests and clinical parameters were assessed.

Due to the lack of clearly stated norms for CPET in the group of children and adolescents, the results of the study group were compared with the results of the comparative group, as well as data available in the literature.

Inclusion criteria for the study group included:

1. Diagnosis of JIA for at least one year prior to commencement of the study.
2. Age between 8 and 17 years.
3. Obtaining the consent of the patient’s legal representatives (parents/legal guardian) to participate in the study.
4. Obtaining the consent of the patient themselves, if, despite being under 16 years of age, they were able to express their opinion on participation in the study with understanding.

The criteria for exclusion from the study group were:

1. Active inflammation in the knee and hip joints and high inflammatory activity in other joints, preventing correct execution of the cardiopulmonary exercise test.
2. Lack of consent from the child’s and/or patient’s legal representatives to participate in the study.
3. Age below 8 years or lack of cooperation of the patient.
4. Active infection.
5. Previously diagnosed cardiovascular and respiratory disorders.

The time criterion was a minimum of 1 year from the diagnosis of the disease. The symptom duration is reported as a median, 25 pc and 75 pc. The source of the data was the patients’ medical records.

Inclusion criteria in the comparison group included:

1. Age between 8 and 17 years, exclusion of cardiovascular and respiratory diseases, matching the study group in terms of age, sex and BMI.
2. Daily physical activity level comparable to the study group (in accordance with WHO recommendations.
3. Obtaining the consent of the patient’s legal representatives (parents/legal guardian) to participate in the study.
4. Obtaining the consent of the patient themselves, if, despite being under 16 years of age, they were able to express their opinion on participation in the study with discernment.

The criteria for exclusion from the comparison group were:

1. Lack of consent from the child’s and/or patient’s legal representatives to participate in the study.
2. Age below 8 years or lack of cooperation from the patient.
3. Active infection.
4. Previously diagnosed cardiovascular, respiratory, or other chronic diseases that could affect the results of cardiopulmonary testing.

In the control group, patient’s medical history was also collected, a survey was conducted regarding their physical activity in accordance with WHO recommendations, a resting 12-lead ECG was performed, and a CPET test was conducted.

The primary objective of the study was to assess the aerobic capacity of children and adolescents with JIA in relation to healthy children in the control group.

The specific objectives included:

1. Assessment of the correlation between aerobic capacity in patients with JIA and disease activity, subtype and duration.
2. Assessment of the impact of treatment, including biological therapy, on the aerobic capacity of patients with JIA.
3. Comparison of ventilation and gas exchange parameters between patients with JIA and the control group.
4. Comparison of ventilation and gas exchange parameters between patients with JIA who engage in physical activity and patients with JIA with a sedentary lifestyle. The study was conducted in accordance with the Declaration of Helsinki, and approved by the Ethics Committee of Medical University of Lodz (protocol nr RNN/200/18/KE from 12 June 2018). Legal guardians of patients consented to review of the medical records and to the use of the data. All the laboratory tests were performed by the standard methods.

### Statistical Analysis

The Shapiro–Wilk test was used for statistical analysis to check the normality of the distribution. Continuous variables are presented as the median with values for the lower and upper quartiles (25th–75th percentile). Categorical variables are presented as numbers with an appropriate percentage. Differences between two groups were assessed using the Mann–Whitney U test. Differences between more than two groups were assessed using the Kruskal–Wallis test. Correlations were assessed using Spearman’s rank correlation coefficient. All *p* values ≤ 0.05 were considered statistically significant. Statistically significant differences between the groups are presented graphically in the charts. Firstly, an analysis was carried out to determine which CPET parameters correlate with age, and not necessarily with JIA. Linear regression was performed in order to check whether the parameters that were previously found to be significant but correlated with age could be independently associated with JIA. Gender in the study and control groups was compared using Pearson Chi2 tests, Chi2 with Yates correction (<15), two-sided Fisher’s exact test (<5). Statistical analysis was performed using Dell Statistica 13.3 data analysis software (StatSoft Polska, Kraków, Poland).

## 3. Results

**Figure 1:**
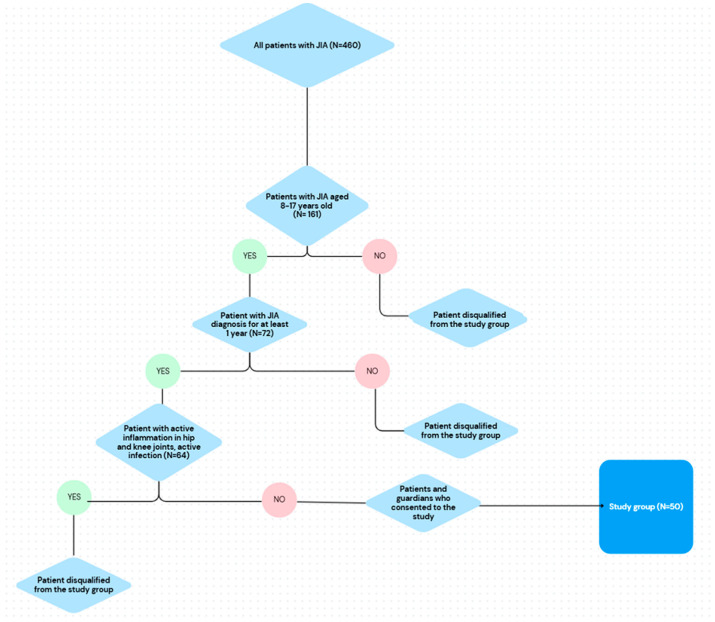
Flowchart representing selection of patients with JIA to the study group.

The study group consisted of 50 patients with JIA (82% female) hospitalized between December 2018 and March 2021. The patient selection process is presented on the flowchart (Figure 1) The median age of the patients was 12.50 years (25 pc = 10.00 years; 75 pc = 16.00 years), and the mean BMI equaled 19.60 kg/m^2^ (25 pc = 17.00 kg/m^2^; 75 pc = 22.10 kg/m^2^). The comparison group consisted of 50 patients and was selected according to age, sex and BMI. Disease activity was assessed using the CHAQ questionnaire and the median score equaled 0.13 points, as well as the JADAS-27 scale, with median of 5.50 points. The exclusion of patients with hip or knee involvement, which potentially indicates higher disease activity, is a limitation of this study and should be evaluated in further studies.

The criterion for inclusion in the study was a minimum diagnosis period of 1 year. In the study group the median diagnosis time equaled 3.75 years (25 pc = 2.00 years; 75 pc = 6.00 years). The duration of the diagnosis was acquired from the records.

Patient fatigue during exercise was assessed using the 10-point BORG scale (mean = 8.00 points). In addition, patients assessed joint pain before and after exercise on a 10-point VAS scale (mean VAS before exercise = 0.72; mean VAS after exercise = 2.02). Characteristics of the study group can be found in Table 1.

At the beginning of the analysis, linear regression was used to see if cardiopulmonary exercise test parameters, which were statistically significant but correlated with age and gender, could be independently associated with JIA. All parameters except exercise load, ventilatory anaerobic threshold, respiratory compensation threshold, and respiratory reserve were statistically significant, meaning that the disease affects the variable independently of age and sex. We have identified that BMI might affect the peakVO_2_.

CPET parameters assessed during the study were: maximal speed, maximal treadmill elevation, load (W), metabolic equivalent (METS), peak oxygen uptake (peakVO_2_), predicted VO_2_max (%), oxygen uptake (VO_2_; L/min), carbon dioxide excretion (VCO_2_; L/min), respiratory exchange ratio (RER), oxygen pulse (O_2_ pulse; mL/beat), minute ventilation (VE; L/min), minute ventilation versus carbon dioxide and its slope (VE/VCO_2_; VE/VCO_2_ slope), ventilatory anaerobic threshold (VAT), respiratory compensation point (RCP), the relationship between VO_2_ and work done per unit time (∆VO_2_/∆WR; mL/min/W), oxygen uptake efficiency ratio (OUES), cardiac output (CO; L/min), resting and maximum heart rate (HR) and heart rate reserve (HRR). Moreover, ventilatory parameters such as maximal voluntary ventilation (MVV), tidal volume (Vt), pulmonary gas exchange (VE/VCO_2_, VE/VO_2_, end-tidal oxygen and carbon dioxide tension (PETO_2_, PETCO_2_)) were evaluated.

All patients achieved RER > 1, with 40 patients achieving RER > 1.2. All patients achieved >85% of maximum heart rate, while 24 patients achieved maximum heart rate. Twenty-four patients achieved RER ≥ 1.2 and HR ≥ 95%, 21 patients achieved RER ≥ 1.1 and HR ≥ 90%, and 5 patients achieved RER ≥ 1.0 and HR ≥ 85%.

**Figure 2:**
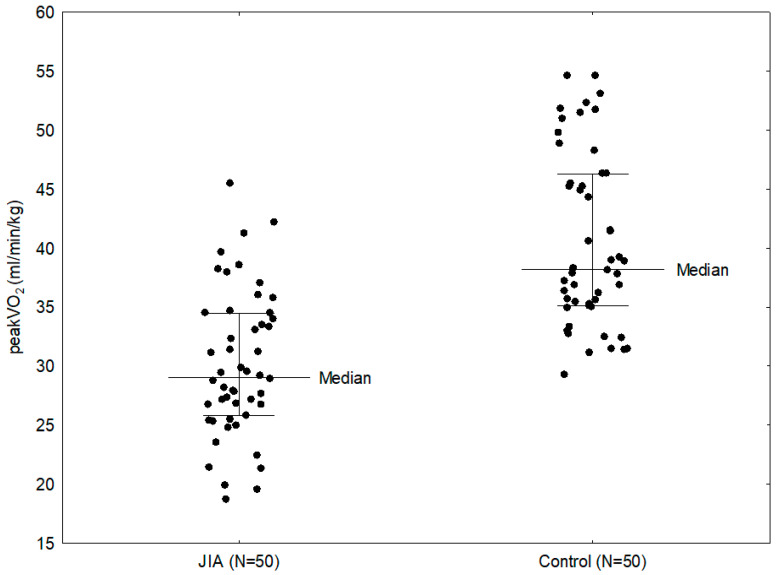
Comparison between peakVO_2_ in the study group and control group.

The study showed that patients with JIA had significantly lower peakVO_2_ compared to patients in the control group (29.05 vs. 38.20 mL/min/kg; *p* < 0.0001) (Figure 2). In addition, the statistically significant parameters in patients with JIA compared to the comparison group were: exercise load (144.00 vs. 223.00 W; *p* < 0.0001), METS (10.20 vs. 13.50; *p* < 0.0001), predicted peakVO_2_ (76% vs. 93%; *p* < 0.0001), VO_2_ (1.37 vs. 2.17 L/min; *p* <0.0001), VCO_2_ (1.66 vs. 2.52 L/min; *p* < 0.0001), O_2_ pulse (7.00 vs. 11.40 mL/beat; *p* < 0.0001), VE (55.50 vs. 84.50 L/min; *p* < 0.0001), VAT (19.45 vs. 23.10 L/min; *p* = 0.0001; 65.5% vs. 59%; *p* = 0.0004), RCP (24.65 vs. 28.70 L/min; *p* = 0.0002; 87.80% vs. 78.50%; *p* = 0.0018), OUES (1.62 vs. 2.17; *p* < 0.0001), CO (8.25 vs. 12.75 L/min; *p* < 0.0001) (Table 2). Children in the study group had significantly shorter exercise times (9:02 vs. 12:00 min), with lower workloads. Children with JIA reached VAT (03:19 vs. 05:16 min) and respiratory compensation threshold (06:46 vs. 08:43 min) significantly earlier with lower peakVO_2_ values than their healthy peers.

Regarding the ventilatory parameters assessed during CPET, additional parameters were considered, including breathing reserve (BR; L/min and %), respiratory rate (RR), maximal voluntary ventilation (MVV; L/min), ventilatory power (VP; mmHg), tidal volume (Vt; L), PETO_2_ and PETCO_2_. Children with JIA had higher BR (43.00 vs. 31.00 L/min; *p* = 0.0002), lower MVV (103.40 vs. 113.60 L/min; *p* = 0.0031) and lower Vt (1.27 vs. 1.67 L; *p* = 0.0002) than patients in the comparison group (Table 3).

CPET parameters were also compared by sex within the study group. Male patients in the study group had a statistically significantly lower peak oxygen uptake compared to female patients with JIA (27.80 vs. 29.20 mL/min/kg; *p* < 0.0001). However, there was higher minute oxygen uptake in the males (2.03 vs. 1.32 L/min, *p* < 0.0001), higher carbon dioxide excretion (2.07 vs. 1.60 L/min, *p* < 0.0001), higher O_2_ pulse (10.10 vs. 7.00 mL/beat; *p* < 0.0001), higher VE (64.00 vs. 54.00 L/min; *p* < 0.0001), higher OUES (2.30 vs. 1.30; *p* < 0.0001), higher CO (12.00 vs. 8.10 L/min; *p* < 0.0001) and VAT (22.90 vs. 20.05 L/min; *p* = 0.0047). Respiratory parameters were also compared, with male patients having higher MVV (118.70 vs. 98.30 L/min; *p* < 0.0001), Vt (1.93 vs. 1.25 l; *p* < 0.0001) and BR (50.00 vs. 42.00 L/min; *p* < 0.0199) than female patients.

**Figure 3:**
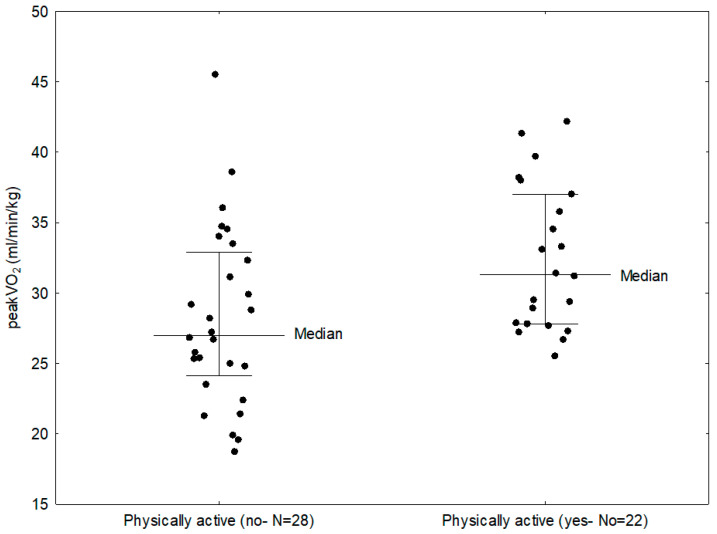
Comparison between peakVO_2_ in children with JIA who were physically active and those who did not exercise.

Twenty-two patients in the study group (44%), have declared exercising according to WHO recommendations. Comparing CPET parameters in these patients and those who did not do any exercise, it was shown that exercising patients had significantly higher maximal oxygen uptake (31.30 vs. 27.00 mL/min/kg; *p* = 0.0099) (Figure 3) and VO_2_/WR (7.35 vs. 5.45 mL/min/W; *p* = 0.0041). The study group was also evaluated according to the subtype of JIA. In the study group, 25 children (50%) were diagnosed with the oligoarticular type of the disease, 13 children (26%) with the polyarticular type, 8 children (16%) with the enthesitis-related arthritis (ERA) and 4 children (8%) with the systemic subtype. In a number of cases systemic subtype progressed to the polyarticular subtype, thus for statistical analysis purpose they were assessed together as a single group. Comparing the three groups (1—polyarticular + systemic, 2—oligoarticular, 3—ERA), it was found that patients with systemic and polyarticular types had lower peakVO_2_ than patients with other types of the disease (28.90 vs. 29.10 vs. 31.15 mL/min/kg); however, this result was not statistically significant. Parameters that showed statistically significant difference between disease subtypes were: VO_2_ (1.31 vs. 1.27 vs. 1.97 L/min; *p* = 0.0056), VCO_2_ (1.60 vs. 1.58 vs. 2.31 L/min; *p* = 0.0057), O_2_ pulse (7.00 vs. 6.95 vs. 11.35 mL/beat; *p* = 0.0041), OUES (1.47 vs. 1.48 vs. 2.21; *p* = 0.0107, CO (8.10 vs. 7.75 vs. 11.60 L/min; *p* = 0.0056); load (154.00 vs. 118.00 vs. 234.00; *p* = 0.0060), MVV (94.50 vs. 97.00 vs. 119.95 L/min; *p* = 0.0149) and Vt (1.25 vs. 1.24 vs. 1.95 L; *p* = 0.0087) (Table 4). The best cardiopulmonary parameters were obtained by patients with ERA subtype, which suggests that the subtype of the disease might have an impact on performance parameters. The differences between the subtypes might be influenced by disease activity, as well as increased inflammation and more aggressive treatment in the systemic and polyarticular subtypes.

In the study group, disease activity assessed with the JADAS-27 scale and the CHAQ, as well as joint ultrasound images, did not correlate significantly with any of the CPET parameters; however, the exclusion of patients with hip or knee involvement, which potentially indicates higher disease activity, is a limitation of this study and should be evaluated in further studies. Similar results were obtained for disease duration and CPET parameters. A lower CHAQ score was shown to correlate with higher minute ventilation (R = −0.35; *p* = 0.0116).

Examining the effect of inflammatory markers on CPET parameters, there was no significant effect of ESR values on any of the studied parameters. However, a negative correlation was observed between CRP levels and VO_2_ (R = −0.35; *p* = 0.0116), O_2_ pulse (R = −0.35; *p* = 0.0127); OUES (R = −0.44; *p* = 0.0015), CO (R = −0.36; *p* = 0.0101); VAT (R = −0.50; *p* = 0.0002), RCP (R = −0.32; *p* = 0.0257), MVV (R = −0.32; *p* = 0.0243) and Vt (R = −0.32; *p* = 0.0256).

In the study group, 38 patients (76%) were treated with systemic corticosteroids. These patients had higher VO_2_ (1.48 vs. 1.18 L/min; *p* = 0.0481) and O_2_ pulse (7.60 vs. 6.00 mL/beat; *p* = 0.0136) compared to patients who did not receive steroids. Forty-four patients (88%) were treated with methotrexate. Treatment with this drug had no significant effect on the CPET parameters. Sulfasalazine was administered to 13 patients (26%). Patients treated with this medication had lower values of VO_2_ (*p* = 0.0124), VCO_2_ (*p* = 0.0070), O_2_ pulse (*p* = 0.0247), VE (*p* = 0.0145), OUES (*p* = 0.0241), CO (*p* = 0.0096), and Vt (0.0145) than patients who did not receive sulfasalazine treatment.

Eighteen patients (36%) received hydroxychloroquine. Compared to patients not receiving this drug, they had a significantly lower peak VO_2_ (26.00 vs. 29.65 mL/min/kg; *p* = 0.0230). Patients receiving biologic agents (tocilizumab—8/50 patients, adalimumab—10/50 patients, etanercept—2/50 patients) did not exhibit any significant changes in CPET parameters. The duration of treatment with each medication also had no significant effect on CPET results in our study group.

We also analyzed the presence of immunological markers of the disease in each patient in the study group and their effect on aerobic capacity. In the study group, one patient (2%) had positive rheumatoid factor, and two patients (4%) had positive anti-CCP antibodies; due to the small number of patients, correlations could not be made for these markers. Positive ANA were found in 22 patients (44%). They had no significant effect on CPET parameters.

## 4. Discussion

According to research conducted over the years, obesity, chronic inflammation and reduced physical activity may have a synergistic effect on cardiovascular risk in patients with JIA. As is known from the literature, physical activity is an important factor in controlling body weight, lowering blood pressure, improving aerobic capacity and musculoskeletal welfare [1]. Despite complex and advanced therapeutic options for the treatment of JIA, which provide improved clinical outcomes and better disease control, complete remission is achieved in only 20–25% of patients [1,16]. Reduced physical activity and performance in children with JIA is likely to have a multifactorial background. It may be the result of active disease or long-term joint damage, musculoskeletal complications, applied treatment (long use of glucocorticosteroids), as well as environmental and psychological influences. Children and adolescents themselves often believe that excessive activity can exacerbate or provoke further joint damage [1,17].

It is well known that children with JIA are less active than their peers and present reduced physical performance [18]. Children with JIA may have reduced aerobic and anaerobic capacity, impaired muscle strength and lower bone density. Suboptimal physical activity and exercise capacity can contribute to further deterioration and disability, putting children with arthritis at risk of declining well-being [19]. Two questionnaires are used to assess the well-being of patients with JIA: CHAQ and the Child Health Questionnaire (CHQ). The CHAQ is a diagnostically specific questionnaire for JIA, translated and validated in Polish, among other languages. The questionnaire refers to the last 14 days and includes eight different activity categories (dressing, eating, walking, standing up, reaching, grasping, hygiene and activity). The total score ranges from 0 (no limitations) to 3 (significant limitations) [20,21]. In our study, the median CHAQ score of the patients was 0.13, meaning that most of them had no limitations in performing daily activities.

WHO guidelines on physical activity and sedentary behavior in children and adolescents state that many benefits are observed with moderate-intensity physical activity of at least 60 min per day. There is evidence that increased aerobic physical activity improves cardiorespiratory fitness and resistance exercise increases muscular endurance and strength in children and adolescents [22]. Recent studies have shown that exercise is safe, well tolerated and beneficial for improving muscle strength, bone mineral density and functional capacity in children with JIA. Current guidelines from the American College of Sports Medicine and a recent review on exercise therapy in children with JIA recommend an exercise program at least 3 days per week [3]. Importantly, a recent Cochrane review on exercise in JIA found no adverse effect of exercise on disease activity. According to the 2002 recommendations of the Exercise and Physical Activity Conference Arthritis Working Group, children with JIA should regularly perform moderate-intensity exercise and muscle-strengthening exercises [1]. A 2008 American Academy of Pediatrics (AAP) guideline on conditions that affect sports participation states that children with systemic or human leukocyte antigen B27 (HLA-B27)-associated JIA require cardiovascular evaluation regarding the potential for cardiovascular complications during exercise, but they are allowed to perform physical activity [23]. Children who are unable to perform aerobic exercise to the desired extent should be encouraged to engage in regular exercise tailored to their clinical condition [24]. Despite the studies available in the literature on the beneficial effects of physical activity on many traditional cardiovascular risk factors in our study group, only 22 patients (44%) performed the physical activity recommended by the WHO.

Physical activity contributes to the proper development of the child, is essential for the optimal metabolic rate of the body, prevents many chronic diseases, and is essential for the social, emotional and cognitive development of children and adolescents. Compared to healthy peers, patients with JIA have significantly lower levels of physical activity, and lower total energy expenditure. Aerobic capacity in children with JIA is 22% lower than that of their healthy peers [1]. In the Netherlands, only 23% of patients with JIA, compared to 66% of healthy peers, complied with recommendations to spend at least 1 h a day on active recreation. Interestingly, low levels of physical activity in adolescents with JIA did not correlate with disease activity and did not increase with better control of disease activity [25]. In the study group, peakVO_2_ was 4.30 mL/min/kg higher in children performing physical activity according to WHO recommendations compared to those patients who did not engage in any physical exercise. It has been proven that children with JIA can benefit from exercise testing and training during the remission period to reduce inflammation and improve aerobic capacity, muscle strength and functional ability, which may be associated with better standard of living [26]. Recent studies suggest that exercise therapy is safe and does not exacerbate the disease [19]. Physical activity affects key mediators of the pathogenesis of this disease (i.e., cortisol, interleukin-6 (IL-6), calprotectin and miRNA-146a). There is a negative correlation between the amount of regular physical activity and plasma IL-6 levels [27]. Adult patients with JIA, despite achieving disease remission, have subclinical evidence of chronic inflammation, as indicated by elevated levels of inflammatory cytokines, markers of endothelial activation and oxidative stress, and adipokines, molecules closely involved in vascular changes. Chronic inflammation is also a well-defined non-traditional risk factor in the pathogenesis of atherosclerosis, where cytokines such as interleukin 1 (IL-1), interleukin 6 (IL-6), and TNF-α may promote endothelial dysfunction, which plays a key role in the process of atherogenesis. These results suggest that long-term inflammation, even during remission, may promote changes in the adipose tissue of these patients, increasing the risk of cardiovascular disease [28]. Currently, there is also much discussion about the role of gut microbiota in the development of JIA, as well as sphingolipids as molecules involved in pathological inflammatory responses [29].

Health-related physical fitness consists of various components, the most important of which are body composition and cardiopulmonary fitness (maximal oxygen uptake). Physical activity is significantly associated with cardiopulmonary fitness in children with JIA [30].

CPET has gained popularity for its complementary value in the diagnostic process and is increasingly used in daily clinical practice. This test evaluates the function of multiple systems which interact to meet increased muscle oxygen demand and the removal of carbon dioxide produced by metabolic processes during submaximal and maximal exercise. The results of this test can be used to help assess disease severity, prognosis, response to treatment and provide important information for clinical decision-making. This test includes measurement of respiratory gas exchange: VO_2_, VCO_2_ and VE, as well as monitoring of electrocardiography, blood pressure and pulse oximetry, usually during a submaximal progressive exercise tolerance test [14].

VO_2_ max, classically referred to as the highest achievable O_2_ uptake for a given individual, is a parameter determined during dynamic exercise based on the “plateau” of VO_2_ despite continuously increasing load. In the absence of a clear plateau, the highest VO_2_ actually achieved in a test is more correctly referred to as peakVO_2_, or the highest VO_2_ achieved in a test performed to the limit of tolerance. This applies to pediatric patients, in whom a plateau of this parameter is difficult to achieve. Both VO_2_max and peakVO_2_ are conventionally expressed in units of milliliters per minute or liters per minute or, after correcting for body weight, as milliliters per minute per kilogram. It has been shown that peakVO_2_p can be reliably assessed during graded intensity exercise (both on a bicycle ergometer and treadmill) in children and adolescents with JIA [31]. In addition to peakVO_2_, physiological criteria, i.e., achievement of adequate HR and RER values at VO_2_max, are used to assess the achievement of maximal effort during pediatric CPET [14]. In our study, achievement of maximal effort was assessed by maximal HR, RER, Borg fatigue score, minute ventilation and minute VE/VCO_2_ slope.

The limitations of achieving maximal exercise have led experts in exercise physiology to develop parameters, such as the OUES. The OUES concept is based on a linear relationship between minute ventilation and VO_2_ during a progressive CPET test. Higher OUES values indicate more efficient VO_2_ absorption [9]. OUES is commonly applied as a marker of heart failure in children with congenital heart diseases and a value of 1.6 was the cut-off point [32]. The median OUES in the study group was 1.62 and 2.17 in the control group (*p* < 0.0001). In physically active patients median OUES was 1.82 vs. 1.53 in patients who led a sedentary lifestyle, however these values were not statistically significant. Subtype of JIA may influence OUES values, with the highest values observed in children with the ERA subtype, and the lowest values in children with systemic and polyarticular JIA (2.21 vs. 1.47, *p* = 0.0107). There was a negative correlation between CRP activity and OUES (*p* = 0.0015), treatment did not significantly affect OUES values.

The respiratory compensation point (AT), also known as the lactate threshold, is considered an indicator of the onset of metabolic acidosis. It is a useful submaximal parameter in children and a good indicator of exercise capacity in children who are unable to achieve maximal effort during exercise. AT occurs at 66% of peakVO_2_ in 8- and 9-year-old children and at about 60% of peakVO_2_ in the older age group [33]. In the study group, the median AT achieved by patients with JIA was within normal range at 65.5% of peakVO_2_.

CO is another parameter that increases during exercise in response to increasing tissue energy demands. CO is the best indicator of cardiac function during exercise. In healthy individuals, CO is a linear function of VO_2_ and does not vary with gender or fitness level [14]. In our study CO was assessed by non-invasive means, through the estimated, indirect Fick method. This method has its limitations in terms of the accuracy of results; however, it allows us to avoid invasive measurement and reduces the risk of, among other things, uncooperative behavior from patients, as with other more invasive methods, thus it is mostly implemented in pediatric centers. Median CO values equaled 8.25 L/min in the study group and 12.75 L/min in the control group (*p* < 0.0001). Comparing the subtypes of JIA, we found the highest CO in patients with the ERA type (11.60 L/min; *p* = 0.0056).

CPET also includes assessment of ventilatory parameters. Regarding pulmonary function tests, both restrictive and obstructive pulmonary impairment have been found in children with JIA, and there is a significant inverse correlation between pulmonary function parameters and rheumatoid factor level, ESR, disease duration and duration of methotrexate therapy [34]. Healthy children in the control group had significantly higher MVV than children with JIA (113.60 vs. 103.40 L/min; *p* = 0.0031) and higher Vt (*p* = 0.0002). There was no statistically significant difference in ventilation parameters in JIA patients who were physically active and those who were not physically active. The highest ventilatory parameters were observed in children with ERA subtype. In addition, negative correlations were found between MVV, Vt and CRP levels (*p* = 0.0243; *p* = 0.0256). In our study we did not confirm the impact of disease activity on these parameters. Patients treated with sulfasalazine had significantly lower VE (*p* = 0.0145). No similar relationship was found in patients receiving methotrexate.

In order to accurately interpret CPET results, it is necessary to refer to age-specific normative values for exercise capacity because of the significant changes in oxygen transfer and consumption that occur during childhood, adolescence and adulthood. These changes are caused by puberty, changes in lean mass and growth-related changes in the ratio of stroke volume and heart rate, which affect cardiac output and oxygen consumption of the exercising muscle [35].

Takken et al. in their review demonstrated that children with JIA show moderate to severe impairment of peakVO_2_p compared to healthy children, indicating a clinically significant reduction in physical capacity [13]. Reduced aerobic capacity is associated with longer disease duration and is likely related to inactivity, reduced fitness, muscle atrophy, or weakness. The relationship between aerobic capacity and disease activity is less clear, with impaired aerobic capacity found in both children with active disease and those in remission [19]. In the study group, we did not find significant correlation between peakVO_2_ and inflammatory markers or disease duration. Regarding the applied treatment, a significantly lower peakVO_2_ was observed in children who received hydroxychloroquine. However, the subtype of the disease and the presence of underlying inflammation might have affected other gas exchange parameters, such as VO_2_, O_2_ pulse and OUES. Patients with the ERA subtype had the highest gas exchange parameters. Patients with systemic and polyarticular onset of the disease had the worst cardiopulmonary results. Although statistical significance was only achieved for VO_2_ and OUES, these results indicate a possibility of subclinical changes in the cardiovascular system in patients with these subtypes of the disease. Due to the limited size of the subgroups the results are exploratory in nature. Worse exercise capacity is often difficult to distinguish from early or mild heart disease. Clinical history, as well as certain CPET parameters can serve as an aid in the evaluation of heart failure. It has been found that in patients with heart failure, there is a lower ratio of VO_2_ to load (ΔVO_2_/ΔWR). The relationship between VO_2_/WR slope is a relatively new parameter used mainly in children with congenital heart defects. The determination of this slope is an important measure of O_2_ flow or utilization in tissues subjected to exercise. It has been found to have a value of about 9.5 mL O_2_/min/W regardless of age [14,33]. VO_2_/WR slope was decreased in the study group, but it was not significantly different from the result obtained in the control group. However, children with JIA performing physical activity had significantly higher VO_2_/WR (7.35 mL/min/W vs. 5.45 mL/min/W; *p* = 0.0041). Peak oxygen pulse, a ratio of VO_2_ to HR reflecting the amount of O_2_ extracted per heartbeat, is usually low in patients with heart failure [14]. In the study group, O_2_ pulse was significantly lower than in the control group (median 7.00 mL/beat vs.11.40 mL/beat; *p* < 0.0001). The lowest O_2_ pulse values were found in children with systemic and polyarticular types of JIA, and the highest in children with ERA subtype (*p* = 0.0041). There was also a negative correlation between CRP levels and O_2_ pulse (*p* = 0.0127). Patients who received corticosteroids had higher O_2_ pulse (*p*= 0.0136). In the study group, 38 patients (76%) were treated with systemic corticosteroids. 16 patients had oligoarticular subtype, 10 patients had polyarticular subtype, 8 patients had ERA subtype and 4 patients had a systemic subtype. In the subgroup of patients who received corticosteroids 28 patients were females, 10 patients were males. Median age of patients who received corticosteroids was 13 years old, median BMI equaled 20.8 kg/m^2^, median disease activity assessed in the JADAS-27 scale equaled 6.52, median duration of the disease was 5.09 years; 16 patients in this subgroup were considered as physically active. The data in this group do not differ significantly from the overall group data, except for a slightly longer time since diagnosis (3.75 years vs. 5.09 years). Given the uneven size of the groups and the fact that the physical fitness of patients could be influenced by the subtype of the disease (assuming a milder course in patients with oligoarticular subtype), the conclusions cannot be considered binding. This is one of the limitations of the study, and statistical analysis on this topic should be expanded in the future. The improved results of certain CPET parameters in patients receiving glucocorticosteroids may be related to the short-term administration of these drugs, which prevents the onset of adverse effects, but may also result from the fact that short-term use of glucocorticosteroids can improve physical performance. As reported by Collomp et al. and Risser et al., short-term glucocorticosteroid administration increases endurance and improves maximal performance and aerobic performance [36,37]. The CPET parameters may have been influenced by the fact that patients who were given a low dose of steroids for a short period of time had lower disease activity and were therefore able to perform increased physical activity. This data must be confirmed in future studies, preferably on larger groups of patients.

Another useful parameter in assessing cardiac efficiency is the VE/VCO_2_ slope. An increase in this slope caused by a decrease in cardiac output relative to metabolic rate is often observed, especially in moderate to severe heart failure. The average minimum VE/VCO_2_ value is approximately 25 in healthy young people. An abnormal VE/VCO_2_ slope (greater than 34) during exercise is suggested to be an independent predictor of mortality in patients with heart failure and often correlates with disease progression [14]. The median VE/VCO2 in the study group was 27 and did not differ significantly from the value obtained in the comparison group.

Acer, in his study involving 32 JIA patients and 11 healthy controls, revealed that JIA patients have lower aerobic capacity; in addition, factors affecting VO_2_ were sex of the patient, knee or hip involvement and CHAQ score [38]. In our study group, median peakVO_2_ was higher in female than male patients (*p* < 0.0001). CHAQ had no effect on peak oxygen uptake, correlating negatively solely with VE (*p* = 0.0116). Patients with polyarticular and systemic forms of the disease had the worst CPET scores, but these were not statistically significant (*p* = 0.8027). The study found a significant difference in median peak VO_2_, which was 29.05 mL/min/kg in JIA patients vs. 38.20 mL/min/kg in healthy patients. In a study by Takken et al. the median peak oxygen uptake in children with JIA was 33.9 mL/min/kg [30]. On the other hand, van Brussel, in his study comparing the capacity of children with JIA with healthy children, obtained a peak oxygen uptake of 34.6 mL/min/kg in JIA patients vs. 49.1 mL/min/kg in healthy children [39], proving that there might be a significant moderate to severe deterioration in oxygen capacity in our patient group.

### 4.1. Future Directions

The group of patients with JIA included in this study is one of the few groups in Europe to have undergone cardiopulmonary exercise testing. All available gas exchange and ventilation parameters were assessed and compared with the results of the control group. In the future, it would be worthwhile to conduct larger, preferably multicenter studies in order to determine the actual level of physical fitness in the population of children with JIA and to correlate the data obtained with the subtype of the disease or the applied treatment. With the introduction of new therapies tailored to specific patients, the development of rehabilitation methods also adjusted to specific patients and their capabilities, and taking into account their limitations, the capacity of patients with JIA should improve. This is also influenced by doctors, psychologists, and dietitians raising awareness about the safety of physical activity and its measurable benefits, especially in patients with chronic diseases such as JIA. In the era of the aforementioned development of nanomedicine, it would also be worthwhile to examine the impact of this treatment on oxygen capacity and the functional state of the circulatory system of patients with JIA. It would be worthwhile for other centers treating large populations of patients with JIA to also conduct such functional and imaging tests of the cardiovascular and respiratory systems following the application of various types of physical rehabilitation and physical activity. This would allow us to draw conclusions and issue recommendations concerning physical activity in these children as well as its safety.

### 4.2. Limitations of the Study

The main limitation of this study is the small sample size, resulting from, among other things, the fact that the study was conducted at a single center. Furthermore, another limitation was the lack of CPET parameter standards in the group of children; patients with chronic diseases should be their own control group by performing subsequent tests; the control group was not ideally matched due to the significant predominance of females in the study group. It was also a cross-sectional study, thus requiring follow-up on a larger study group. Exclusion of patients with hip or knee joint involvement is a limiting factor in the study, as most of them, despite being unable to perform physical activity on a treadmill, had exacerbated disease and potentially more severe JIA, which could affect CPET parameters. The small number of patients’ systemic and polyarticular subtypes limited the possibilities for statistical analysis for these two independent groups, thus the results of the subgroups are exploratory in nature. Patients were not assessed on the Tanner scale and based on their lean body mass, which is a limitation of the study and should be considered in further research. Another limitation of the study was the assessment of the patient’s physical activity based on a questionnaire, which limits the reliability of such data. Follow-up studies will be conducted using wearable devices to measure the actual duration of participants’ physical activity, their heart rate range during activity, and the type of physical exercise they perform. Moreover, variables such as age, gender, and BMI might have affected patients’ physical performance, thus a more accurate statistical assessment should be carried out in the future.

## 5. Conclusions

1. Patients with JIA have reduced aerobic capacity compared to healthy children.
2. Disease subtype may affect patients’ aerobic capacity. Disease duration might not significantly affect the aerobic capacity of patients with JIA. The impact of disease activity on CPET parameters is unclear; further research on larger patient groups is necessary to verify this.
3. The applied treatment might affect the physical fitness of patients with JIA, as well as their cardiopulmonary parameters. No significant effect of biological treatment on improving the aerobic capacity of patients with JIA was observed.
4. Patients with JIA have significantly lower values for most gas exchange and ventilatory parameters in comparison to the control group.
5. Cardiopulmonary exercise testing might be considered a useful tool for assessing and monitoring the oxygen capacity of patients with JIA, and some of its parameters may be useful in assessing the risk of cardiovascular complications in this group of patients.
6. Patients with JIA who are physically active have significantly higher aerobic capacity, and therefore exercise programs used as adjuvant therapy should be recommended to patients with JIA and adapted to their abilities and the number of joints affected by the disease.

## Figures and Tables

**Table 1:** General characteristics of the study group.

	N	(%)
Sex (male)	9	18.00
Feature	N	Me (25–75%)
Age (years)	50	12.50 (10.00–16.00)
BMI (kg/m^2^)	50	19.60 (17.00–22.10)
CHAQ	50	0.13 (0.00–0.38)
PDUS	50	0.00 (0.00–1.00)
JADAS-27	50	5.50 (1.00–10.00)
Duration of illness (years)	50	3.75 (2.00–6.00)
Disease subtype	N	(%)
Systemic onset	4	8.00
Polyarticular RF−	12	24.00
Polyarticular RF+	1	2.00
Oligoarticular	25	50.00
ERA	8	16.00
Immunological/genetic test results	N	%
ANA (positive titer)	22	44.00
RF (positive)	1	2.00
HLA-B27 (positive)	13	26.00
anti-CCP (positive)	2	4.00

BMI—body mass index, CHAQ—Childhood Health Assessment Questionnaire, PDUS—power Doppler ultrasound, JADAS-27—Juvenile Arthritis Disease Activity Score, RF—rheumatoid factor, ERA—enthesitis-related arthritis, ANA—antinuclear antibody, HLA-B27—human leukocyte antigen B27, anti-CCP—anti-Cyclic Citrullinated Peptide. Variables are presented in the form of a median with the lower and upper quartiles (25–75 percentile).

**Table 2:** Comparison of CPET results between patients with JIA and a control group.

		JIA	Control	
Number of patients		50	50	
Characteristic	Unit	Me (25–75%)	Me (25–75%)	*p* value *
Velocity max.	km/h	6.20 (5.40–6.70)	6.80 (6.70–8.00)	**<0.0001**
Elevation max.	%	15.10 (14.00–16.00)	16.10 (16.00–18.00)	**<0.0001**
peakVO_2_	mL/min/kg	29.05 (25.80–34.50)	38.20 (35.10–46.30)	**<0.0001**
predicted VO_2_	%	76 (63–91)	93 (84–99)	**<0.0001**
VO_2_	L/min	1.37 (1.12–1.83)	2.17 (1.66–2.86)	**<0.0001**
VCO_2_	L/min	1.66 (1.30–2.07)	2.52 (1.95–3.24)	**<0.0001**
RER	-	1.19 (1.11–1.26)	1.20 (1.15–1.25)	0.5713
O_2_ pulse	mL/beat	7.00 (6.00–9.10)	11.40 (8.90–15.10)	**<0.0001**
VE	L/min	55.50 (44.00–66.00)	84.50 (72.00–98.00)	**<0.0001**
VE/VO_2_	L/min	26.26 (24.34–28.91)	25.35 (23.33–27.28)	0.1973
slope VE/VCO_2_	-	27.25 (23.70–30.30)	26.70 (25.10–29.10)	0.8794
OUES	-	1.62 (1.22–2.05)	2.17 (1.68–3.26)	**<0.0001**
CO	L/min	8.25 (6.80–11.00)	12.75 (10.20–16.50)	**<0.0001**
VE/VCO_2_	L/min	27.00 (26.00–30.00)	27.00 (26.00–29.00)	0.3213
Load	W	144.00 (103.00–203.00)	223.00 (177.00–273.00)	**<0.0001**
MET	-	10.20 (10.00–12.95)	13.50 (13.30–17.10)	**<0.0001**
VAT	L/min	19.45 (16.80–22.30)	23.10 (20.20–24.90)	**0.0001**
VAT	%	65.50 (59.00–77.00)	59.00 (52.00–68.00)	**0.0004**
RCP	L/min	24.65 (21.60–29.10)	28.70 (25.50–35.90)	**0.0002**
RCP	%	87.80 (79.00–92.00)	78.50 (70.00–84.00)	**0.0018**
VO_2_/WR	mL/min/W	5.90 (4.70–8.40)	6.05 (5.10–8.60)	0.4218
HRR	L/min	14.00 (6.00–22.00)	12.50 (6.00–20.00)	0.6365

peakVO_2_—peak oxygen uptake, VO_2_-oxygen uptake, VCO_2_—carbon dioxide excretion, RER—respiratory exchange ratio, O_2_ pulse—oxygen pulse, VE—minute ventilation, OUES—oxygen uptake efficiency ratio, CO—cardiac output, MET—metabolic equivalent of task, VAT—ventilatory anaerobic threshold, RCP—respiratory compensation point, VO_2_/WR—the relationship between VO_2_ and work done per unit time, HRR—heart rate reserve * significant *p*-value < 0.05. The differences between the 2 groups were assessed using the Mann–Whitney U test. Continuous variables are presented in the form of a median with the lower and upper quartiles (25–75 percentile). The values presented in bold are statistically significant.

**Table 3:** Comparison of respiratory parameters between JIA and a control group.

		JIA	Control	
Number of patients		50	50	
Characteristic	Units	Me (25–75%)	Me (25–75%)	*p* value *
BR	(L/min)	43.00 (32.00–56.00)	31.00 (19.00–38.00)	**0.0002**
BR	(%)	44.00 (33.00–54.00)	29.00 (18.00–38.00)	**<0.0001**
Bf	(L/min)	41.50 (35.00–50.00)	46.00 (41.00–51.00)	0.0546
MVV	(L/min)	103.40 (80.40–117.00)	113.60 (103.40–125.10)	**0.0031**
VP	(mmHg)	4.84 (4.22–5.60)	5.26 (4.50–6.08)	0.0584
Vt	(L)	1.27 (1.04–1.69)	1.67 (1.46–2.24)	**0.0002**
PETO_2_	(mmHg)	110.00 (107.00–113.00)	111.00 (108.00–114.00)	0.2598
PETCO_2_	(mmHg)	34.00 (32.00–35.00)	33.00 (31.00–35.00)	0.3798

BR—breathing reserve, Bf—breathing frequency, MVV—maximal voluntary ventilation, VP—ventilatory power, Vt—tidal volume, PETO_2_—end-expiratory partial pressure of oxygen, PETCO_2_—end-expiratory partial pressure of carbon dioxide. * Significant *p*-value *p* < 0.05. The differences between the two groups were assessed using the Mann–Whitney U test. Continuous variables are presented in the form of a median with the lower and upper quartiles (25–75 percentile). The values presented in bold are statistically significant.

**Table 4:** Comparison of CPET results between patients with different types of JIA.

JIA Subtype		Systemic and Polyarticular	Oligoarticular	ERA	
Number of patients		17	25	8	
Characteristic	Units	Me (25–75%)	Me (25–75%)	Me (25–75%)	*p* value *
peakVO_2_	(mL/min/kg)	28.90 (25.50–33.50)	29.10 (26.05–34.50)	31.15 (27.85–34.65)	0.8027
VO_2_	L/min	1.31 (1.14–1.63)	1.27 (1.09–1.52)	1.97 (1.73–2.69)	**0.0056**
VCO_2_	L/min	1.60 (1.17–1.90)	1.58 (1.14–1.75)	2.31 (2.00–3.06)	**0.0057**
RER	-	1.20 (1.15–1.26)	1.18 (1.10–1.27)	1.19 (1.10–1.28)	0.6753
O_2_ pulse	mL/beat	7.00 (5.90–9.55)	6.95 (5.90–8.35)	11.35 (8.45–14.40)	**0.0041**
VE	L/min	55.00 (46.00–61.00)	50.00 (42.50–62.50)	83.00 (66.50–91.50)	**0.0149**
VE/VO_2_	L/min	25.33 (23.93–27.72)	26.63 (24.88–29.42)	27.07 (22.63–29.79)	0.5631
OUES	-	1.47 (1.11–1.83)	1.48 (1.29–1.93)	2.21 (1.80–2.60)	**0.0107**
CO	L/min	8.10 (6.40–9.90)	7.75 (6.70–8.95)	11.60 (10.30–15.70)	**0.0056**
HR max.	Beats/min	193.00 (187.00–200.00)	200.00 (189.00–203.00)	195.50 (186.50–204.50)	0.7266
Load	W	154.00 (97.00–203.00)	118.00 (91.00–180.00)	234.00 (169.50–306.50)	**0.0060**
VO_2_/WR	mL/min/W	5.20 (4.20–8.40)	5.95 (4.90–8.60)	7.25 (5.80–8.55)	0.4839
HRR	L/min	14.00 (6.00–22.00)	14.00 (5.50–21.50)	13.50 (5.00–29.50)	0.9450
MVV	L/min	94.50 (80.40–113.60)	97.00 (79.15–113.00)	119.95 (109.75–144.55)	**0.0149**
Vt	L	1.25 (0.99–1.63)	1.24 (1.08–1.47)	1.95 (1.48–2.33)	**0.0087**
BR	L/min	43.00 (26.00–59.00)	43.50 (33.50–52.50)	38.00 (32.50–65.50)	0.9843
Bf	L/min	44.00 (35.00–54.00)	41.00 (34.50–48.00)	38.00 (34.00–48.50)	0.6237

peakVO_2_—peak oxygen uptake, VO_2_-oxygen uptake, VCO_2_—carbon dioxide excretion, RER—respiratory exchange ratio, O_2_ pulse—oxygen pulse, VE—minute ventilation, OUES—oxygen uptake efficiency ratio, CO—cardiac output, HR max.—maximal heart rate, VO_2_/WR—the relationship between VO_2_ and work done per unit time, HRR—heart rate reserve, MVV—maximal voluntary ventilation, Vt—tidal volume, BR—breathing reserve, Bf—breathing frequency. * Significant *p*-value < 0.05. The differences between the >2 groups were assessed using the Kruskal–Wallis test. Continuous variables are presented in the form of a median with the lower and upper quartiles (25–75 percentile). The values presented in bold are statistically significant.

## Data Availability

Data available on request due to restrictions (privacy, pediatric patients).

## Abbreviations

The following abbreviations are used in this manuscript:

JIA	Juvenile Idiopathic Arthritis
RA	Rheumatiod arthritis
peakVO_2_	Peak oxygen uptake
WHO	World Health Organization
CPET	Cardiopulmonary exercise test
CRP	C- reactive protein
ESR	Erythrocyte sedimentation rate
RF	Rheumatoid factor
ANA	Anti-nuclear antibodies
CHAQ	Childhood Health Assessment Questionnaire
VAS	Visual Analogue Scale
BMI	Body mass index
RER	Respiratory exchange ratio
HRmax	Maximum heart rate
JADAS-27	Juvenile Arthritis Disease Activity Score-27
W	Load
METS	Metabolic equivalent
VO_2_	Oxygen uptake
VCO_2_	Carbon dioxide excretion
O_2_ pulse	Oxygen pulse
VE	Minute ventilation
VE/VCO_2_	Minute ventilation versus carbon dioxide
VAT	Ventilatory anaerobic threshold
RCP	Respiratory compensation point
∆VO_2_/∆WR	The relationship between VO_2_ and work done per unit time
OUES	Oxygen uptake efficiency slope
CO	Cardiac output
HR	Heart rate
HRR	Heart rate reserve
Vt	Tidal volume
BR	Breathing reserve
RR	Respiratory rate
MVV	Maximal voluntary ventilation
VP	Ventilatory power
ERA	Enthesitis- related arthritis
CHQ	Child Health Questionnaire
AAP	American Academy of Pediatrics
HLA-B27	Human leukocyte antigen B27
IL-6	Interleukin-6
